# Effect of Public Expenditure on Economic Growth in the Case of Ethiopia

**DOI:** 10.1155/2023/9305196

**Published:** 2023-02-01

**Authors:** Girma Mulugeta Emeru

**Affiliations:** Department of Economics, Debre Berhan University, P.O. Box 445, Debre Berhan, Ethiopia

## Abstract

This study's primary goal was to explain how Ethiopia's economic growth affected government spending. The time series data utilized in the study were gathered between 1980 and 2018. The time series data were subjected to the Johansen cointegration test and the vector error correction model (VECM) in order to evaluate the short- and long-term correlations between public spending and economic growth in Ethiopia. According to the study, both long- and short-term economic growths are positively and significantly impacted by government spending on education. Long-term economic growth is negatively impacted by government expenditure on agriculture, while short-term effects are negatively impacted and considerable. In the long run, investment spending has a positive but negligible impact on economic growth; however, in the short run, it has a negative but large effect. Defense spending by the government has a positive and negligible effect on economic growth over the short and long terms. Both in the short and long terms, spending on health has a favorable and considerable impact on economic growth. According to the study, government spending on the education sector would help to foster the conditions that could result in higher labor force participation rates and, consequently, higher rates of economic growth. Aiming to establish a healthy and productive society that promotes economic progress, policy should focus on complementary measures to scale-up initiatives in the health sector.

## 1. Introduction

Following Nigeria in terms of population, Ethiopia has the second-fastest expanding economy on the continent, with 6.3% growth in FY2020/21. However, with a gross national income of $960 per individual, it is also among the poorest [1]. As the most significant macroeconomic indicator of a society's overall performance, economic growth is the outcome of creating more goods and services, which calls for increased worker productivity and labor supply expansion. The government must invest resources to uphold contracts, preserve national security, deter crime, and provide valued public goods in order to ensure that markets function properly and spur economic progress [2].

The government of Ethiopia has a major responsibility to supply the country's 84.8 million citizens with public goods and services such as defense, communication, energy, roads, agriculture, and food security (MOFED [3]). According to MOFED (2013), 29.6% of Ethiopia's population is below the poverty line, and the country's average GDP per person is $550 (MOFED, 2014). Since the 19th century, there have been numerous discussions and analyses of the relationship between government spending and economic growth in the economic literature. As much as the daily, it has been investigated in numerous studies pertaining to this topic. Two ideas developed by Wagner and Keynes in their respective theoretical works are the connection between government spending and economic growth. Keynes defended the claim that increased public spending is the result of increased economic growth, which is what Wagner contends [4].

Currently, it is crucial to research how Ethiopia's economic growth is impacted by the breakdown of government spending. This research adds to the body of knowledge by providing further insight into how public spending affects economic growth in Ethiopia. The study makes three contributions to the body of existing literature. First, there have been several debates concerning the connection between government spending and economic expansion. According to several empirical studies, there is a strong and positive correlation between government spending and economic growth [5–9], while many empirical investigations indicate a strong negative link [10–13]. In addition, there are several empirical research [14–17] studies whose findings are conflicting, while in others there is no correlation between government spending and economic growth, for instance [18]. The goal of this study is to add to the body of knowledge on Ethiopia's economic development, not to resolve long-standing disputes and arguments concerning the impact of government spending. Second, as governments become more accountable and involved in the economy, public spending is rising quickly in populous nations like Ethiopia. The studies that attempt to analyze the effects of public sector spending on the nation's economic growth are scarce in Ethiopia, and the majority of them are out-of-date ([19–22], and [23] are just a few examples). Third, this study notably varies from earlier research studies in that it was conducted from 1980 to 2018 a pretty lengthy time period. Therefore, the goal of this study is to investigate, using the Johansen cointegration test and the vector error correction model, the impact of public spending on economic growth in the case of Ethiopia (VECM).

The study was organized into five parts in which the first part of the study deals with the introduction; the second part, deals with review of the related literature; the third part, deals with research methodology; the fourth part, deals with result and discussion and the final part of the study deals with conclusion and recommendation.

## 2. Literature Review

### 2.1. Empirical Studies

Scholars have conducted a variety of research studies to better understand the relationship between public spending and economic growth, agrioutput, education, health, investment, and inflation.  Table 1 lists various relevant empirical literatures.

## 3. Research Methodology

### 3.1. Data Description and Source

This study tries to determine how Ethiopia's government spending components affect economic growth. Additionally, the study plans to employ secondary time series data for the years 1980–2018 that were gathered from various sources. Because reliable data were available for the majority of the variables used in this particular study, the researcher used data from the World Bank's World Development Indicators. As a result, information about total government spending on education, health, agriculture, defense, and investment was gathered from the World Bank and MOFED, while information about the consumer price index (CPI) was gathered from the National Bank of Ethiopia (NBE) and the World Bank.

### 3.2. Methods of Data Analysis

For better understanding and discussion, the secondary data was examined using a description and econometric model. The primary benefit of the VECM is that it has a beautiful interpretation with long-term and short-term equations. Additionally, VECM is only a representation of a cointegrated VAR; therefore, the researcher utilized VECM to examine the existence of a long-run and short-run link between the variables. The debate and findings served as the foundation for the conclusion and, finally, the recommendation.

### 3.3. Model Specification

The mathematical explanation of the relationship between the dependent and independent variables is referred to as model specification. With a focus on sectorial spending, this section provides an econometric model for the relationship between government spending and economic growth in Ethiopia. Government investment spending was a further pertinent variable employed in this study, and the consumer price index (CPI) served as a control variable. The empirical framework of this study uses the VAR approach to assess the relationship between government spending on the areas of agriculture, defense, health, education, and investment and economic growth in Ethiopia. Because there is a bidirectional relationship between government spending and economic growth, the VAR technique is used. The evidence remains as to whether the supply of public goods and services leads to economic growth or whether economic growth drives the demand for goods and services.

(1) $$\begin{array}{c} \begin{array}{c} GDPGROWTH=f\left(public\;ExpenditureComponents\right)\text{,}\\ GDP\;GROWTH=\frac{GDP\;per\;Capitat-GDP\;per\;Capita\;t-1}{GDP\;per\;Capita\;t}\text{.} \end{array} \end{array}$$

The public expenditure components which include: Health, Education, Military and Infrastructure expenditures are in million Ethiopian Birr per year. The proportion of the expenditure share to the total GDP for each expenditure component was calculated as:

(2) $$\begin{array}{c} Expenditure\;Share\;t=\frac{Expenditure\;t}{GDP\;t} \end{array}$$

Hence the theoretical model is modified to be of the form:

(3) $$\begin{array}{c} GDPGROWTH=f\left({ExpenditureShare}_{t}\right)\text{.} \end{array}$$

From the forgoing discussion, the composition of government expenditure is an important determinant of growth. Thus the model express GDP as a function of various components of government expenditure that include total expenditure on agriculture, investment, defense, education, health sectors and in addition consumer price index (CPI) was included, since it can be have a lasting impact on economic growth.

The functional relationship between dependent and independent variables is shown as follows; Thus, the growth model is specified as follows:

(4) $$\begin{array}{c} \text{RGDP}=F\left(\text{gexpedu},\text{gexphe},\text{gexpagri},\text{gexpdef},\text{gexpinv},\text{CPI},\text{Ui}\right)t\text{,} \end{array}$$

where *t* is the time, RGDP is the real gross domestic product, gexpeduis the total expenditure on education, gexphe is the total expenditure on health, gexpagri is the total expenditure on agriculture, gexpdef-the total expenditure on defense, gexpinv is the total expenditure on investment, CPI is the Consumer Price Index, and Ui is the error term.

All model variables are in real terms which are captured by dividing each nominal quantity by general price index (CPI). Expressing the dependent variables in logarithm form, an attempt were have been made to examine the impact of each explanatory variable on economic growth.

In log-linear form, the model is specified as follows:

(5) $$\begin{array}{c} \log\;RGDP=B0+B1\;\log\;gexpedu+B2\;\log\;gexphe+B3\;\log\;gexpagri+B4\;\log\;gexpde\;f+B5\;\log\;gexpinv+B6\;\log\;CPI+Ui\text{,} \end{array}$$

where; log (RGDP) is the logarithm of real GDP, log (gexpedu) is the logarithm of total government expenditure on education, log (gexphe) is the logarithm of total government expenditure on health, log (gexpagri) is the logarithm of total government expenditure on agriculture, log (gexpdef) is the logarithm of total government expenditure on defense, log (gexpinv) is the logarithm of total government expenditure on investment, log (CPI) is the logarithm of consumer price index, Ui is the error term and from *B*_0_, and *B*_1_–*B*_6_ are the parameters.

In prior estimation of the growth model mentioned above, standard econometrics tests such as stationary and cointegration tests were conducted in order to avoid the generation of spurious regression results. The researcher also used vector error correction model (VECM) to analyze both the short and long run impact of sectorial expenditure on agriculture, defense, investment, education, and health of economic growth.

### 3.4. Definition and Measurement of Variables

  Real gross domestic product (RGDP): this is the percentage rate of increase in gross domestic product. It captures the changes in value of goods and services produced in a given economy for a specific period of time. It will be calculated as a percentage rate of change of the GDP Ademuyiwa and Adetunji [34].
  Public expenditure on education (gexpedu): this is the share of expenditure in education to total government expenditure. It includes the expenditure government incur to fund basic up to higher education, by paying for teachers and lecturers, construction of learning infrastructure such as class rooms, lecture halls, offices and purchasing of learning equipment. It also includes expenses on scholarships whether local or abroad [34].
  Public expenditure on health (gexphe): this is the share of public expenditure on health to total government expenditure. It contains the amount government spends on hospital building structures, equipping the hospital institution with equipment and drugs, training of doctors and nurses and paying their salaries [35].
  Public expenditure on agriculture (gexpagri): this is the share of total government expenditure on agriculture. It includes expenses such as buying modern agricultural equipment, agricultural inputs such as improved seeds, trained and hiring a number of agricultural development agents and so on [36].
  Public expenditure on defense (gexpdef): this is the fraction of expenditure on defense against the gross government expenditure. It includes expense such as buying military gadgets and equipment, salaries, training the defense forces, supporting missions and operations and expense for facilitating wars [22].
  Public expenditure on investment (gexpinv): it is provided by government total capital expenditure less capital spending on health and education and the Government itself has been investing heavily to improve the country's infrastructures. The Government is also focusing on the housing sector and low cost condominiums are being built in the capital and regional cities. Moreover, the Government is actively engaged in an initiative to improve the ease of doing business in Ethiopia [37].
  Consumer Price Index (CPI): it is a measure that examines the weighted average of prices of a basket of consumer goods and services or it is the measure of the average change in prices over time that consumers pay for the basket of goods and services [38].  Table 2 shown below.

### 3.5. Time Series Analysis

The study used time series econometric models in establishing the relationship between GDP growth and Public expenditure components, as a share of GDP. The linearity relationship is assumed between variables for the model specified in Section 3.3 above. To address the objective of the study, the data was analyzed step by step using the processes and methods as described in the proceeding sections.

#### 3.5.1. Stationarity Test

Stationarity of a series is important due to influence it has on its behaviour. Thus, if *X* and *Y* series are nonstationary processes, then modelling *X* and *Y* relationship as a simple linear regression as in (8) shown below will lead to spurious regression [39].

(6) $$\begin{array}{c} Yt=\beta_{0}+\beta_{1}X_{t}+\varepsilon_{t}\text{.} \end{array}$$

Time series data are said to be stationary if its mean, variance, and covariances do not vary overtime. Non-stationary data leads to spurious regression due to non-constant mean and variance [40]. Differencing a series using differencing operators produces other set of observations. For instance, the first-differenced values are given as: Δ*X*_*t*_ = *X*_*t*_ − *X*_*t*_ − 1. If a series is stationary without any differencing, it is said to be *I* (0) or integrated of order 0. However, if a series is stationary after first difference is said to be *I* (1) or integrated of order 1. In order to check for stationarity in the series (whether in levels or first-differences), the Dickey and Fuller (1979) test was used.

#### 3.5.2. Cointegration Test

After establishing whether the series is stationary in levels or first difference (and if the series are integrated of the same order), then Johansen's procedure is used to determine whether there exist a cointegrating vector among the variables [41]. The procedure uses two tests to determine the number of cointegrating vectors which are the maximum eigenvalue test and the trace test. The null hypothesis for the maximum eigenvalue is to test *r* cointegrating relations against the alternative of *r* + 1 cointegrating relations where *r* = 0, 1, 2,…, *n* – 1, and *n* is the number of variables in the system. The test statistic for maximum eigenvalue is computed as

(7) $$\begin{array}{c} LR_{\max}\left(\frac{r}{n}+1\right)=-T*\;\log\;\left(1-\hat{\omega }\right)\text{,} \end{array}$$

where *ω* is the maximum eigenvalue and *T* is the sample size.

The trace statistics tests the null hypothesis of *r* cointegrating relations against the alternative of *n* cointegrating relations, where *n* is the number of variables in the system and *r* = 0, 1, 2,…, *n* − 1. The test statistic is computed using the following expression:

(8) $$\begin{array}{c} LR_{tr}\left(r/\right)=-T*\overset{n}{\underset{i=r+1}{\sum }}\log\;\left(1-\omega \hat{i}\right)\text{.} \end{array}$$

In some cases the maximum eigenvalue statistic and the trace statistic may yield different results. In the event that this happens, Alexander [42] indicates the results of the trace test should be preferred.

#### 3.5.3. Vector Error Correction Model (VECM)

After the Johansen cointegration test is performed, the next step is to fit the appropriate time series model. If cointegration has been established between the variables, then this implies that there exists a long-run relationship between the variables. Hence, the VECM is applied in order to determine the short-run relationships of cointegrated variables. On the other hand, if there is no cointegration, then the VECM is reduced to a vector autoregressive (VAR) model, and the Granger causality tests will be used to determine causal links between the variables. The regression equation form for VECM is given as

(9) $$\begin{array}{c} \Delta GDPgrowth_{t}=\alpha_{1}+\alpha_{1}e_{t-1}+\overset{n}{\underset{i=0}{\sum }}\beta i\;\Delta GDPgrowth_{t-i}+\overset{n}{\underset{i=0}{\sum }}\;\delta i\Delta ExpShareX_{t-i}\Delta ExpShare_{t}=\alpha_{1}+\alpha_{1}e_{t-1}+\overset{n}{\underset{i=0}{\sum }}\beta i\Delta {ExpShare}_{t-i}+\overset{n}{\underset{i=0}{\sum }}\delta i\;\Delta {GDPgrowthX}_{t-i}\text{ .} \end{array}$$

In the VECM modeling above, the cointegration rank shows the number of cointegrating vectors. For example, a rank of four indicates that four linearly independent combinations of the nonstationary variables will be stationary. The error correction component (ECM), given as the coefficient of *e*_t−1_, shows the speed of adjustment of the variables towards a long-run equilibrium after short run fluctuations of the variables.

### 3.6. Diagnosis Test

To estimate a more specific relationship between public expenditure and economic growth, it is very important to establish whether the estimation coefficients are tenable and whether the extent to the regression coefficients fitted in the model makes the model a liner, unbiased estimator of the impact of government expenditure on economic growth. The model would be tested to verify the existence of autocorrelation and stationarity effects, which are mostly common in time series data.

#### 3.6.1. Testing Stationary

To estimate a more specific relationship between economic growth and components of government expenditure, the researcher is sure that the time series data are stationary. Most economic data are nonstationary (random walk). There exists a trend element in which both independent and dependent variables grow upward or decrease downward continuously together [43].

The common tests used are the Dickey–Fuller (DF) and augmented Dickey–Fuller (ADF) tests. These tests are basically required to ascertain a number of times variables have to be differentiated to arrive at stationarity. Time series data are said to be differentiated of order “*p*” if it became stationary after differentiating it “*p*” times. Economic variables stationary from the outset are *I* (0) series which are variables that require to be differentiated once to be stationary *I* (1).

#### 3.6.2. Autocorrelation Test

Autocorrelation is a correlation between members of a series observation ordering in time (as time series data). The classical linear regression model assumes that the disturbance (error) term relates to any observation. In running OLS estimation, the most important assumption is that the consecutive error terms are not correlated or that there is no autocorrelation. Running OLS estimation by disregarding autocorrelation will result in inefficiency in the estimated result, and its standard errors are estimated in the wrong way [43]. There are two detecting or testing mechanism formal method like Breusch–Godfrey (BG) test and the Durbin–Watson *d* test and informal method like graphic method.

## 4. Results and Discussion

### 4.1. Descriptive Statistics

Annual time series data from 1980 to 2018 are utilized in this study. Gross domestic output, total government spending on defense, health, education, investments, agriculture, and consumer price index are the factors being taken into account. The other variables are determinant factors of the dependent variable, real gross domestic product (RGDP), whereas RGDP is a dependent variable (see Table 3).

The description of the variables used in the estimation is shown in the above table. They all used millions of Ethiopian Birr. The researcher can observe from the table that the range of the RGDP is 107,221.2 to 909,388.1 million ETB, with a standard deviation of 218.912 million ETB, and the average is 281,767.3 million ETB. Defense spending ranges from 634 to 9,080 million ETB, with an average of 3,121.875 million ETB. The average agricultural spending in the nation is 4,383.655 million ETB, which ranges between 138.294 and 18,999.32 million ETB and a standard deviation of 5,939.871 million ETB. With a mean of 2,340.495 million ETB and a range of 74.491 to 13,641.89 million ETB, the country's health spending has a standard deviation of 5,397.067. The average of educational expenditure is 12,141.33 million ETB and varies from 2,162.109 to 44,560.02 million ETB, with a standard deviation of 12,095.89 million ETB. The average investment expenditure of the country is 3,395.563 million ETB and varies from 17.2942 to 64,343.64 million ETB, with a standard deviation of 10,251.59 million ETB. Finally, the mean of consumer price index is 69.8045 million ETB and varies from 12.92441 to 270.0568 million ETB, with a standard deviation of 74.11584 million ETB. Due to the assumption that their contribution to growth becomes high with a minimum percentage of total expenditure going to defense, the nation's highest maximum expenditure in this case is going to the main sectors of investment, education, agriculture, and health.

### 4.2. Trend of Economic Growth

**Figure 1:**
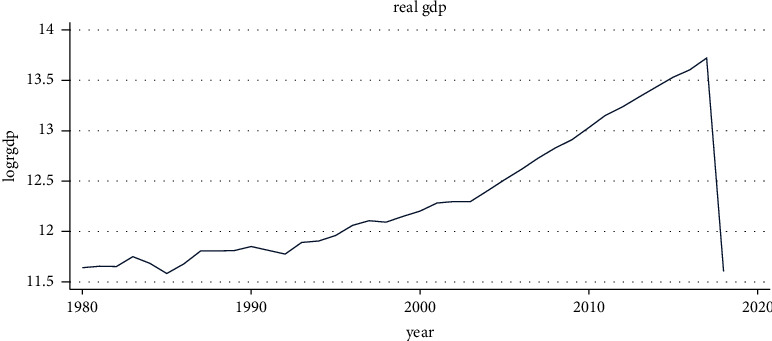
Trend of economic growth. Source: own computation based on the data from MOFED and National Bank, 2022.

As seen in Figure 1, the long-term trajectory of real gross domestic product, up until the middle of the 1980s, it fairly declined. Following that, it began to rise until the end of the 1980s before declining once more in the 1990s. This drop in real gross domestic product was caused by a fierce power struggle between the Ethiopian government and the EPDRF party during this period. The economy is suffering because of the war. Finally, sustained ongoing economic expansion characterizes the time since the 2000s.

#### 4.2.1. Trend of Defense Expenditure and Real Gross Domestic Product

**Figure 2:**
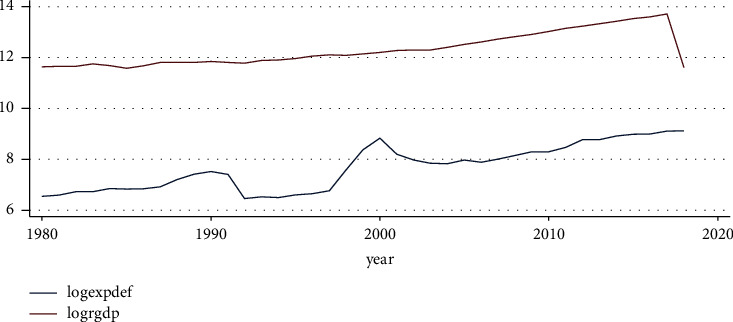
Trend of expenditure on defense and RGDP. Source: own computation based on the data from MOFED and National Bank, 2022.

As can be seen from the graph showing the long-term trajectory of defense spending in Figure 2, it increased slightly up until the 1990s before beginning to decline in the second half of the 1990s. Then, it began to increase dramatically until it reached the year 2000, after which it began to fall sharply. Finally, since the year 2000, defense spending has been steadily rising.

#### 4.2.2. Trend of Agricultural Expenditure and Real Gross Domestic Product

**Figure 3:**
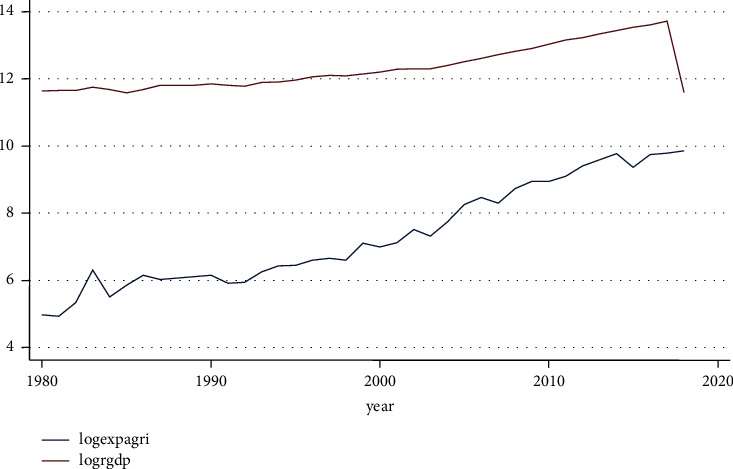
Trend expenditure on agriculture and RGDP. Source: own computation based on the data from MOFED and National Bank, 2022.

Figure 3 shows the long-term trend in agricultural spending..

#### 4.2.3. Trend of Health Expenditure and Real Gross Domestic Product

**Figure 4:**
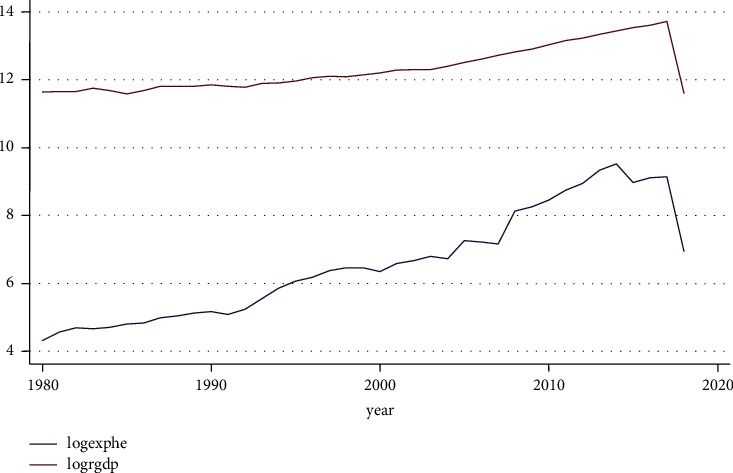
Trend of health expenditure and RGDP. Source: own computation based on the data from MOFED and National Bank, 2022.

Figure 4 shows the long-term trend in health spending. It increased until the second half of the 2000s at a dropping rate before beginning to decline again and then at a rising again.

#### 4.2.4. Trend of Educational Expenditure and Real Gross Domestic Product

**Figure 5:**
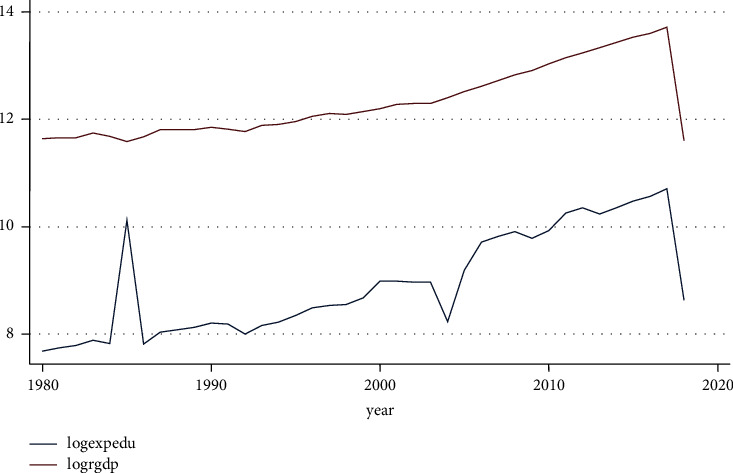
Trend educational expenditure and RGDP. Source: own computation based on the data from MOFED and National Bank, 2022.

Figure 5 shows a graph of the long-term trend in educational spending. It increased at a consistent rate until the 1990s, at which point it began to decline until the 2010s, when it began to rise again.

#### 4.2.5. Trend of Investment Expenditure and Real Gross Domestic Product

**Figure 6:**
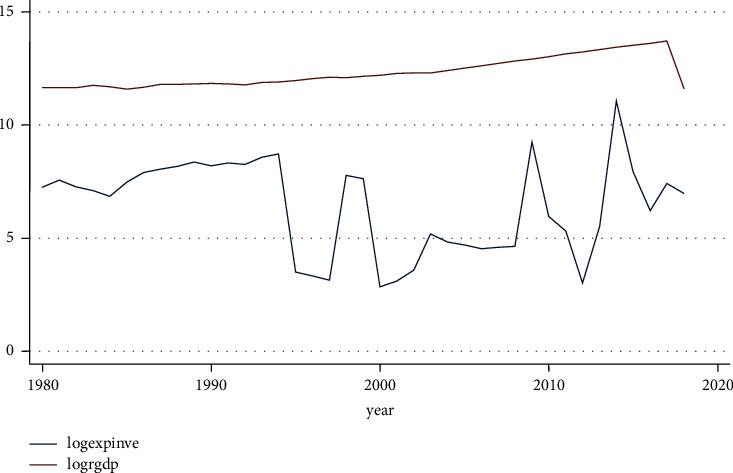
Expenditure on investment. Source: own computation based on the data from MOFED and National Bank, 2022.

Figure 6 shows the long-term trajectory of investment expenditure. It continued to decline gradually up until the second half of the 1990s, after which it started to decline at an ever-increasing rate until it reached 2000. After that, the graph's wave took on an up-and-down kink, but between 2000 and 2010, it significantly declined.

#### 4.2.6. Trend of Consumer Price Index and Real Gross Domestic Product

**Figure 7:**
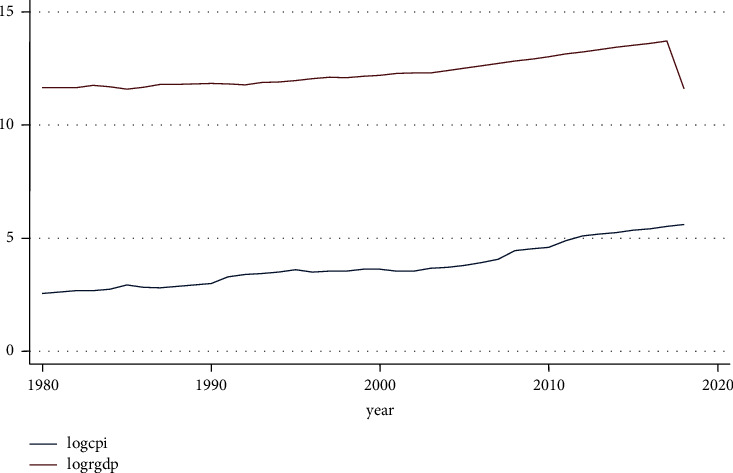
Trend of consumer price index and RGDP. Source: own computation based on the data from MOFED and National Bank, 2022.

As evident from the graph of the Consumer Price Index's long-term trend in Figure 7, up until the year 2000, it began to grow at a decreasing rate before beginning to grow at an increasing rate.

### 4.3. Econometrics Result

#### 4.3.1. Testing for Stationarity

Time series plots usually give the simplest method for checking the stationarity of the variables in the dataset. These findings show that the first difference of the variables has to perform to achieve stationarity of the variables.  Table 4 presents the results of the ADF unit root test for the first difference of the variables (without trend). The results show that all variables are stationary after the first difference, implying that the variables are integrated to order one-*I* (1).

#### 4.3.2. Diagnostic Test of the Model

Diagnostic tests are executed on the model after all the estimations have been run and the models have been specified. Unexpectedly, the model broke the homoscedasticity assumption and encountered the heteroscedasticity problem, which is typical for cross-sectional data but rare for time-series data. A re-estimation of that model was carried out by including a phase to address the heteroscedasticity problem. In addition to the aforementioned problems, other diagnostic tests demonstrate that the diagnostic test's null hypothesis cannot be rejected, indicating that the tested assumptions have not been violated. For instance, the results of the normality test indicate that the null hypothesis cannot be rejected, indicating that the assumptions under test were not violated. It turns out that the assumptions of no autocorrelation, which are supposed to be broken, turn out to be unviolated. Additionally, the presumption of no misspecification is true. Furthermore, diagnostic tests for ARCH and structural break problems show that the null hypothesis cannot be ruled out, suggesting that ARCH does not exist and that the parameters are stable.

#### 4.3.3. Cointegration Analysis

In order to investigate the long-run relationship between GDP growth and public expenditures in health expenditure (gexphe), agricultural expenditure (gexpagri), defense expenditure (gexpagri), educational expenditure (gexpedu), investment expenditure (gexpinv), and Consumer Price Index (CPI), the variables have to first be tested for cointegration. If the series are cointegrated, then the corresponding error correction term and an error correction model must be constructed. The Johansen Cointegration Test is used to determine if the variables co-move towards a long-run equilibrium. The results in Table 5 suggest that the variables are cointegrated. These findings indicate that the appropriate model to fit in the data is VECM.

#### 4.3.4. Vector Error Correction Model

From the cointegration test results presented in Table 5, the following cointegrating relationships were obtained using the Johansen test of cointegration. The equation is solved through the Johansen test, which was used to confirm the appropriateness of the selected equation. To investigate the long-run effects in this model, the researcher presented the estimated normalized cointegration coefficient vectors in Table 6.

From Table 6, the cointegrating equation between GDP growth and public expenditure components (Agriculture, Health, Education, Military, Investment, and Consumer Price Index) for Ethiopia in the period of 1980–2018 is given as

(10) $$\begin{array}{c} \log\;\left(RGDP\right)=8.94+0.026\;\log\;\left(gexpde\;f\right)-0.097\;\log\;\left(gexpagri\right)+0.516\;\log\;\left(gexphe\right)+0.129\;\log\;\left(gexpedu\right)+0.022\;\log\;\left(gexpinve\right)-0.210\;\log\;\left(CPI\right)\text{.} \end{array}$$

With the evidence from the cointegration test, it can be interpreted that economic growth in Ethiopia significantly depends on public expenditure on education and health in the long-run. But the relationship between economic growth and public spending on agriculture has a negative and insignificant impact, and also its relationship with educational spending is positive and significant. The results show that in the long-run, expenditure on investment has a positive coefficient and insignificant impact on economic growth. In addition to this, the Consumer Price Index is negative and insignificant.

The sectorial expenditure on agriculture, investment, defense, and the consumer price index was insignificant. In line with the result of Liu and Li [44], expenditure on agriculture has a negative impact on Ethiopia's economic growth. The reason is that the agricultural sector in Ethiopia has faced a lot of challenges like inadequate finance, poor transportation, inadequate farm inputs, and a lack of land tenure securities over the past few years. These challenges led to a decrease in productivity and the standard of living of rural farmers. This is because the lack of complementary policies, political instability, unexpected shock and natural hazard, corruption, rent seeking problem by officials, having unmotivated civil servants, and poorly administered huge projects result in the unproductive and poor performance of the sector's contribution to growth. This result is similar with the previous studies such as [38, 45, 46] and [47]. But it is against Menyah and Wolde-Rufael [19] and Garoma and Bersisa [48]. However, expenditure on education and health has a positive and statistically significant impact on economic growth. This may be due to the fact that our country is on the way to declining the health condition and substituting the illiterate society with that of the literate by increasing the accessibility of education to create a healthier and more productive society by producing important medicines domestically through the Medical Corporation (MC) and building the medical capabilities of the country's educated force through identifying existing and potential needs based on research and development. The health and educational institution is an umbrella organization for multiple institutions, organizations and factories, and become a leading and an emerging institution in registering remediable growth in the area of manufacturing, agriculture and industrial production, hence the contribution of these institution directly improve the real economic growth of the country, indirectly both have a positive impact on economic growth or it promote growth by increasing the gross domestic product.

The other factor that affects GDP in Ethiopia is expenditure on agriculture. It has a negative long-run impact, a result similar to Teshome [38], Garoma and Bersisa [48], and Bazezew and Alemu [49]. The insignificant and negative long-run effect of agriculture is especially expected from the perspective of economic theory, policy and strategy of the Ethiopian government, the share of agriculture to GDP in the long run should decline and hence investment on agriculture also should be decreased and probably transformed in to industrial economy. The government of Ethiopia has a strategy that says agriculture leads the economy in the short run by creating favourable conditions for industry to play a key role and lead the economy in the long run.

From the above long run equation economic growth with respect to government expenditure changes is highly elastic with a 1% change in government expenditure on health sector leading to an increase in economic growth by 5.2% and also government expenditure is highly elastic with a 1% change in government expenditure on educational sector leading to an increase in economic growth by 13%. The argument of endogenous growth theories of additional effects of human capital over the static effect on the level of output explains sustainable economic growth. But, the coefficient of government expenditure on defense and investment was found to be positive, and Consumer Price Index and agriculture were found to be negative, and all are insignificant. From Stata's results, most of the signs researchers expect are mammalian, with the exception of agricultural and defense signs.

The model estimates that the short-run dynamics are mainly driven by lagged real GDP, total government expenditure on education, investment, health, agriculture, defense, and consumer price index sectors. The short-run coefficients of individual variables should be examined to determine the relative contribution of each component of government expenditure to economic growth in Ethiopia. As shown in Table 7 the coefficient of the first lagged value of real gross domestic product was positive and insignificant. This indicates, in the short run, that real gross domestic product in the current period is not sensitive to what it was in the previous period.

As to health and education expenditure, the coefficient is positive and significant, implying that a 1% increase in education and health spending would lead to an increase in economic growth of 4.593 and 6.171%, respectively, in the short run. This may be due to the fact that when the nation becomes literate and healthier, it may build confidence in the government and the society for both private and government investments in which they may contribute to the GDP of the nation, and it may also avoid fear of instability and further damage to the nation's property because the people are educated and healthier. This also directly improves the economic growth or it helps growth by strengthening the current account balance of the nation by exporting educated and healthier people.

However, the coefficient of the first lagged value of expenditure on investment and agriculture was observed to be negative and significant in the short run. The primary reason for these contrary responses pushes us to look into the components of agriculture spending. Salary for the development agents and recurrent expenditure in the sector are very dominant. In such circumstances, the expenditure on the sector may not help the growth of the economy. The coefficient of the first lagged value of expenditure on investment also has significantly negative effect on economic growth in the short run this shows that the government of the country even spend large amount of capital result in no real economic growth this is because of corruption, access of logistic, lack of good management, lack of cooperation, shortage of basic infrastructure. On the other hand, the coefficient of the first lagged values of expenditure on defense has an insignificant effect on economic growth in the short run.

The coefficient of the error correction term (ECT*t* − 1) for the economic growth equation is significant and positive that is correctly signed and indicates the existence of a long-run relationship amongst the growth model variables. This guarantees that although economic growth may temporarily deviate from its long run equilibrium value, it will gradually reach to its equilibrium after a shock. This implies that in the event of a deviation between the actual and long-run equilibrium levels, there would be an adjustment back to the long-run relationship in subsequent periods to eliminate this discrepancy. The coefficient of the error term and/or the speed of adjustment towards equilibrium value are 0.1727, which implies that there is a relatively high speed of adjustment towards long-run equilibrium. This indicates that whenever there was a disturbance and/or a shock in the system, 17.27% of the deviation or the discrepancy of the actual economic growth from its equilibrium value is eliminated within a year and/or if there is a one percent disequilibrium or shock in the preceding period, the impact of a shock to change in real GDP is corrected by 17.27% per annum.

## 5. Conclusion and Recommendation

The objective of this paper is to investigate the impacts of specific government sectorial spending on economic growth. Using a time series data from the years 1980 to 2018, the researcher investigated the growth impact of government sectorial expenditure on health, agriculture, defense, education, investment, and the consumer price on economic growth in Ethiopia. Before estimating the model, the series was first tested for stationarity and cointegration. After indicating the presence of the long-run relationship using Johansen cointegration approach, the short run dynamics of the long run economic growth is examined by estimating an error correction model.

The researcher finds that in the short-run the main determinant of economic growth is spending on agriculture, education, health, and investment during the study period. On the other hand, the cointegration analysis indicated that the main driving forces behind long-run growth are spending on education and health, while spending on defense and the consumer price index become insignificant both in the short and long-run. Total government expenditure on defense does not have a significant effect on the economy of the country both in the short-run and long-run.

On the other hand, expenditure on health has a positive impact on economic growth both in the long-run and short-run periods and it is also significant both in the long-run and short-run. Moreover, the result shows that expenditure on education contributes to increase in the economic growth. Education not only contributes to economic growth but also the general development of society. In this regard, the researcher's main finding is that education and health are the key sectors in which public expenditure should be directed in order to foster economic growth in the long run.

This study recommended that the government of Ethiopia should spend on human capital (education sector) that contributes significantly to economic growth. In addition, government expenditure on health has a positive and significant effect on economic growth in both the short and long run; therefore, government should increase spending on the health sector because expenditure in these sectors improves the health status of the people and enhances economic growth in the country.

## Figures and Tables

**Table 1:** Empirical literature review.

Author (year)	Location	Method	Result
Meng, 2022 [24]	China	Spearman correlation	The results show that the impact of increasing government financial public health investment on regional economic growth rate is not only related to regional economic aggregate and development level, but also related to regional education level and regional publicity level in logical analysis

Zhou et al., 2021 [25]	China	Generalized method of moments (GMM)	The results indicated that there was an inverted U-shaped relationship between China's institutional environment and its green growth. That is, the institutional environment can initially promote China's green growth but, if it is not changed, will eventually inhibit it

Shabbir et al., 2021 [26]	Pakistan	Autoregressive distributed lags (ARDL)	The long-run findings indicate that foreign private investment has a negative and insignificant impact on economic growth, whereas, domestic investment shows a statistical significance but positive impact on Pakistan economy. The short-run dynamic designates that both domestic and foreign private investment is significantly and positively associated with the growth rate

Li et al., 2021 [27]	Provinces in China	Spatial econometric models	Government public health spending and regional economic growth have significant positive spatial correlation and spatial agglomeration effects. The indicator of government public health spending significantly promotes regional economic growth. In addition, it significantly promotes the economic growth of neighboring areas through certain spatial spillovers

Anwar et al., 2020 [28]	Pakistan	Auto regressive distributed lag modelling technique	Results of the study revealed inflation negatively related to economic performance and positively linked to investment and tradeopenness

Aslam et al., 2019 [29]	Pakistan	Auto regressive distributed lag modelling technique	Empirical outcomes corroborated that government development expenditures had significant positive impact on capital formation in Pakistan. The impact of government expenditures on economy is seemed to be a very important element for economic progression in democratic countries. This study result indicates that it is vital for the Pakistani government to adopt the policy of increase in government expenditures in order to attain higher capital formation, a precondition for growth and development of the country

Rauf et al., 2017 [30]	Pakistan	ARDL method	It is found that the individual impacts of fiscal transfer are although insignificant but still support the theoretical proposition regarding fiscal decentralization and public services relationship while delegation of expenditure responsibilities helps in improving the gross enrollment at primary school level. Furthermore the study evident that complete delegation of fiscal responsibilities to lower governments enhance enrollment ratio in Pakistan

Mukhtarov et al., 2020 [31]	Azerbaijan	ARDLBT, DOLS, and CCR	The estimation results show that government's expenditures on education, gross capital formation and total population have a positive and statistically significant impact on economic growth in the long run

Dehning et al., 2016 [32]	Azerbaijan	ARDLBT approach	Estimation results provide statistically significant and positive contribution of all expenditure items, supported by Keynesian theory. However, productivity of all type of expenditures has significantly decreased after the oil boom

Al‐Abri et al., 2018 [33]	Saudi Arabia	GCC approach	The results suggest that remittance outflows have a weak effect, if at all, on government spending, which, in turn, has an insignificant impact on GDP

**Table 2:** Definition and classification of variables.

Description of variables	Definition of variables	Type of variables	Expected sign
RGDP	The percentage rate of increase in gross domestic product	Continues dependent	—
Education (gexpedu)	The share of expenditure in education to total government expenditure	Continuous independent	+ve
Health (gexphe)	The share of public expenditure on health to total government expenditure	Continues independent	+ve
Agriculture (gexpagri)	The share of total government expenditure on agriculture	Continues independent	+ve
Defense (gexpdef)	The fraction of expenditure on defense against the gross government expenditure	Continues independent	−ve
Investment (gexpinv)	It is provided by government total capital expenditure to improve the country's infrastructures	Continues independent	+ve
Consumer Price Index (CPI)	The consumer price index is a measure that examines the weighted average of prices of a basket consumer goods and services	Continues independent	−ve

**Table 3:** Descriptive statistics of the economic variables (1980–2018 millions of ETB).

	RGDP	expdef	expagri	exphe	expedu	expinve	CPI
Mean	281767.3	3121.875	4383.655	2340.495	12141.33	3395.563	69.8045
Maximum	909388.1	9080	18999.32	13641.89	44560.02	64343.64	270.0568
Minimum	107221.2	634	138.294	74.491	2162.109	17.2942	12.92441
Std. dev.	218912	2649.578	5939.871	3597.067	12095.89	10251.59	74.11584

Source: own computation based on the data from MOFED and National Bank, 2022.

**Table 4:** Augmented-dickey fuller test results at first difference.

Variable	ADF statistics	Critical value	Order of integration
*D* (log RGDP)	−3.528	1% = −3.66 5% = −2.66 10% = −2.616	Stationary at 1^st^ difference
*D* (log expdef)	−5.184		Stationary at 1^st^ difference
*D* (log expagri)	−8.212		Stationary at 1^st^ difference
*D* (log exphe)	−4.276		Stationary at 1^st^ difference
*D* (log expedu)	−6.400		Stationary at 1^st^ difference
*D* (log expinve)	−9.47		Stationary at 1^st^ difference
*D*2 (log CPI)	−7.253		Stationary at 2^nd^ difference

Source: own computation based on the data from MOFED and National Bank, 2022.

**Table 5:** Cointegration test result.

Hypothesized no. of CE(s)	Eigen value	Trace statistics	0.05 critical value	Prob^*∗∗*^
None		135.2957	124.24	0.000
At most 1	0.69703	91.1134	94.15	0.325
At most 2	0.60560	56.6887	68.52	0.311
At most 3	0.42614	36.1400	47.21	0.515
At most 4	0.38182	18.3441	29.68	0.103
At most 5	0.23681	8.3449	15.41	0.004
At most 6	0.15830	1.9687	3.76	0.658
At most 7	0.05182			0.930

Source: own computation based on the data from MOFED and National Bank, 2022.

**Table 6:** Vector error correction model.

Variable	Log (gexpdef)	Log (gexpagri)	Log (gexphe)	Log (gexpedu)	Log (gexpinve)	Log (CPI)	Cons
Coefficient	0.0259	−0.097	0.516	0.129	0.022	−0.210	8.938
T-stat	(0.39)	(−1.18)	(7.48)	(2.04)	(1.61)	(1.85)	

Source: own computation based on the data from MOFED and National Bank, 2022.

**Table 7:** Short run estimation of the VECM model.

Variables	*D* (log RGDP) *t* − 1	*D* (log gexpdef) *t* − 1	*D* (log gexpagri) *t* − 1	*D* (log gexphe) *t* − 1	*D* (log gexpedu) *t* − 1	*D* (log gexpinve) *t* − 1	*D* (log CPI) *t* − 1	ECT (*t* − 1)
Coeff	0.0121	8.765	−30.636	6.171	4.593	−1.749	−118	0.17
*P*-value	(0.252)	(0.412)	(0.001)	(0.000)	(0.005)	(0.048)	(0.38)	(0.0526)

Source: own computation based on the data from MOFED and the National Bank,

## Data Availability

The datasets and articles used to support this study are available from the corresponding author upon reasonable request.
